# First report of a gallbladder hemangioma coexisting with gallstones: a case report and literature review of a rare finding

**DOI:** 10.1186/s12893-022-01554-7

**Published:** 2022-04-06

**Authors:** Giulia Trucco, Luigi Chiusa, Francesco Tandoi, Luca Bertero

**Affiliations:** 1 Pathology Unit, Department of Medical Sciences, University of Turin, Via Santena 7, 10126 Torino, Italy; 2 General Surgery 2U, Department of Surgical Sciences, AOU Città Della Salute e Della Scienza di Torino, Turin, Italy

**Keywords:** Gallbladder, Hemangioma, Gallstones

## Abstract

**Background:**

Gallbladder hemangioma is an exceptionally rare entity, with only ten cases reported in literature hitherto. The here described case is the first report of a gallbladder hemangioma coexisting with gallstones.

**Case presentation:**

A 76-year-old male was hospitalized following repeated episodes of epigastric pain. Patient’s medical history included primary hypertension, type 2 diabetes mellitus, dyslipidemia, obesity and hyperuricemia. Physical examination revealed marked pain in the right hypochondriac region, and laboratory workup was notable for mildly elevated glycemia (125 mg/dL) and pancreatic amylase (60 IU/L). Abdominal ultrasound showed multiple gallstones, a thickened gallbladder wall and mild edema of the perivisceral adipose tissue as well as a hepatic angioma. During surgery, an incidental subserosal nodule of about 1 cm was detected within the gallbladder fundus. After surgery, the clinical course was uneventful and the patient was discharged. Histopathological examination of the subserosal nodule showed multiple dilated vascular channels within a sclerosing matrix, a finding consistent with a cavernous hemangioma. Diffuse chronic cholecystitis was also present.

**Conclusions:**

Gallbladder hemangiomas represent a rare, likely underdiagnosed condition which can be undetected during the preoperative workup.

## Background

**Fig. 1:**
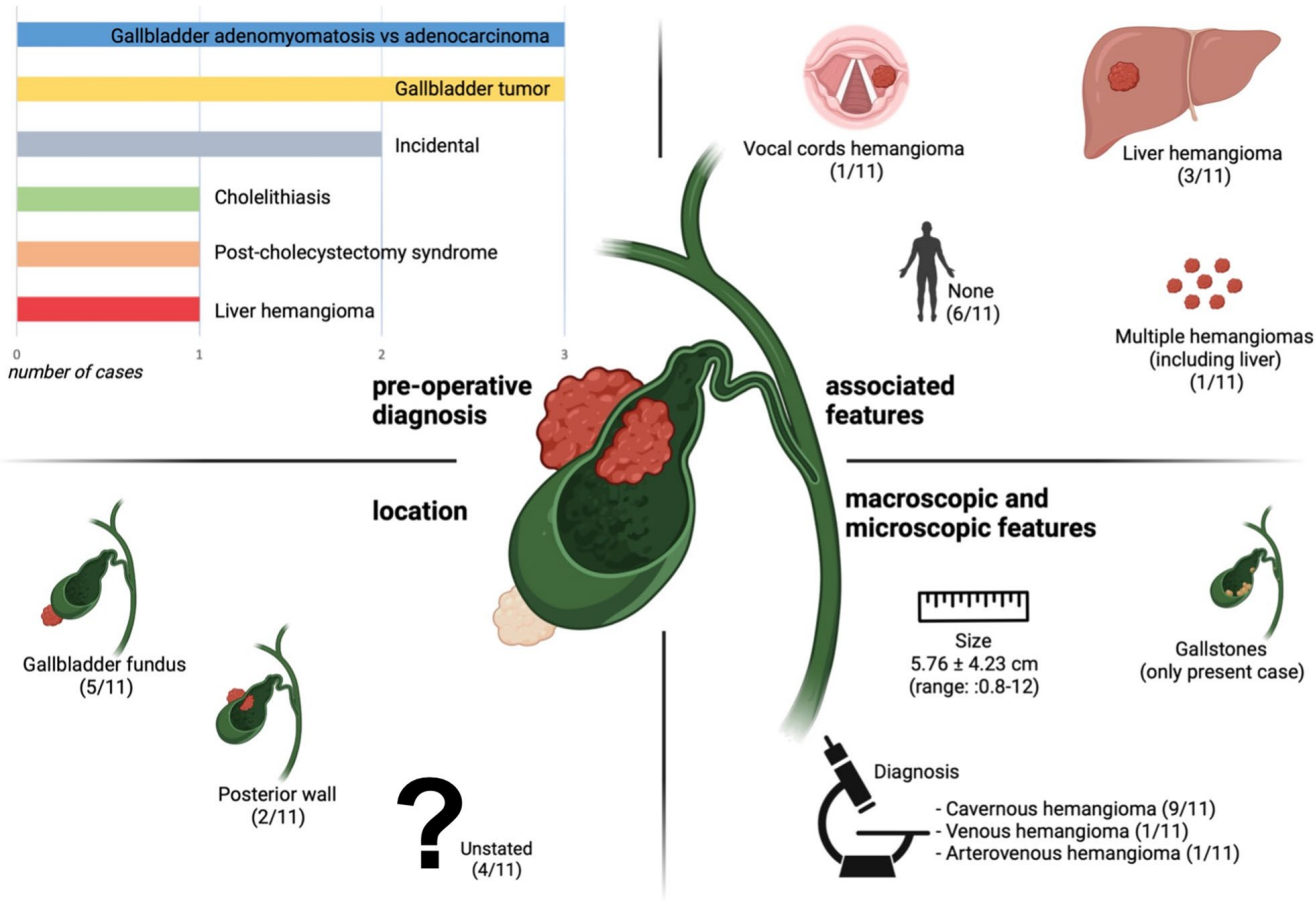
Summary of gallbladder hemangiomas reported to date. Data regarding pre-operative diagnosis, hemangioma location, associated features and macroscopic/microscopic features of all previously reported cases [9–18] are presented. Source: Figure 1 was created using BioRender.com.

Hemangiomas are the most common benign vascular tumors*.* Although ubiquitous, they usually occur as superficial, frequently cutaneous, lesions [1], and about 60% of these lesions are located in the head and neck region [1, 2]. In internal organs, hemangiomas have been mostly reported in liver, where they represent one of the most common benign neoplasms, occurring in 5% of the general population [3]. Conversely, gallbladder hemangiomas are exceptionally rare, with only ten cases reported in literature hitherto (Fig. 1). We here describe the first case of a gallbladder hemangioma coexisting with gallstones.

## Case presentation

**Fig. 2:**
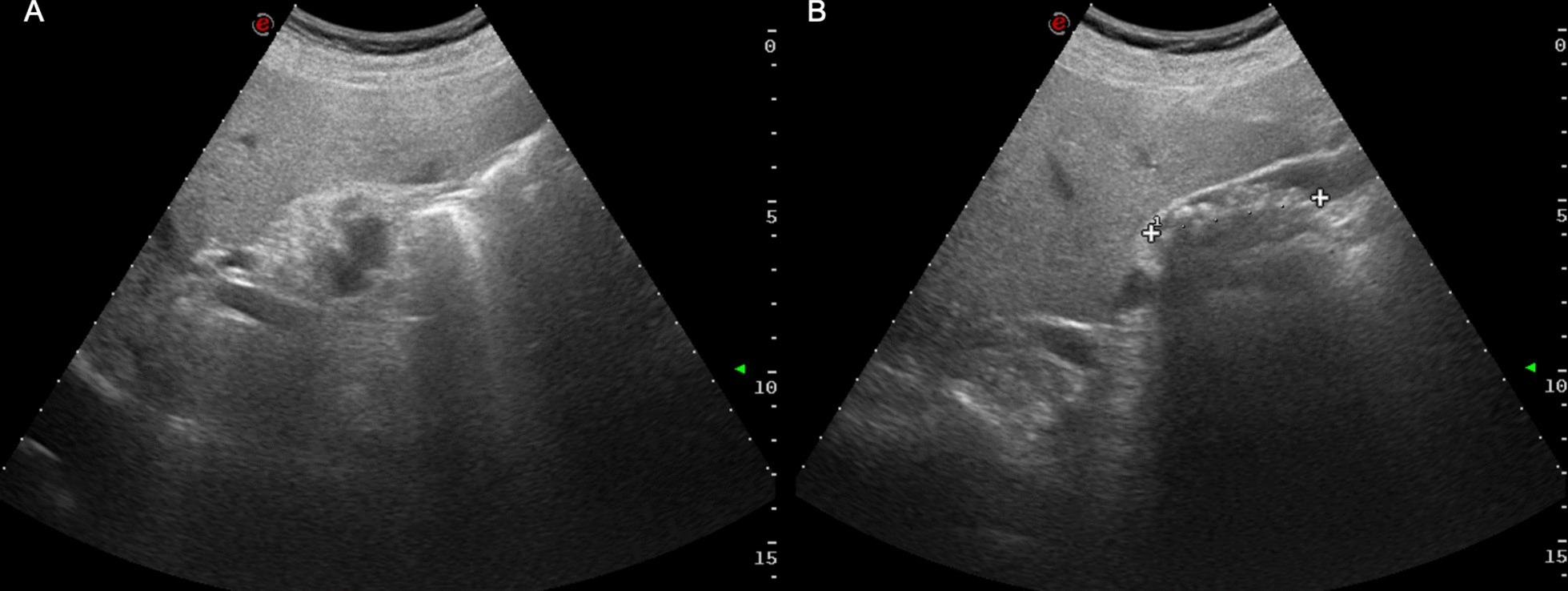
Pre-operative abdominal ultrasound. The dimensions of the liver are at the upper limit of normal, and it presents with rounded margins and diffusely increased echogenicity, findings diagnostic for hepatic steatosis. In this background, in the seventh liver segment, there is a hypoechoic area, measuring 12 mm in diameter, suggestive of hepatic hemangioma (Panel **A**). The intra-hepatic and extra-hepatic biliary ducts are normal. The gallbladder contains a very small amount of liquid, and its lumen is filled by a lithiasic agglomerate, measuring 5 mm in its maximal diameter (Panel **B**). The gallbladder walls are mildly thickened, with a concomitant mild edema of the perivisceral adipose tissue

A 76-year-old male was hospitalized following repeated episodes of heartburn and epigastric pain. Patient’s medical history included primary hypertension, type 2 diabetes mellitus, dyslipidemia, obesity and hyperuricemia. Physical examination revealed marked pain in the right hypochondriac region, and laboratory workup was notable for mildly elevated glycemia (125 mg/dL) and pancreatic amylase (60 IU/L). Abdominal ultrasound showed multiple gallstones, a thickened gallbladder wall with mild edema of the perivisceral adipose tissue, and a hypoechogenic area measuring 12 mm in the seventh liver segment consistent with a hepatic angioma (Fig. 2). Abdominal and chest radiographies were unremarkable. A laparoscopic cholecystectomy was thus performed. During surgery, an incidental finding of a subserosal nodule within the gallbladder fundus was made and the sample, after *en-bloc* removal, was submitted to our department for histopathological examination. Clinical course after surgery was uneventful and the patient was discharged.

Macroscopically, the lesion appeared as a well-defined grayish nodule, measuring 0.8 cm × 0.5 cm, protruding from the gallbladder fundus surface. Nodule consistency was increased, without alterations of the overlying serosa. Gallstones were identified in the gallbladder lumen, but no other macroscopic alterations were found.

**Fig. 3:**
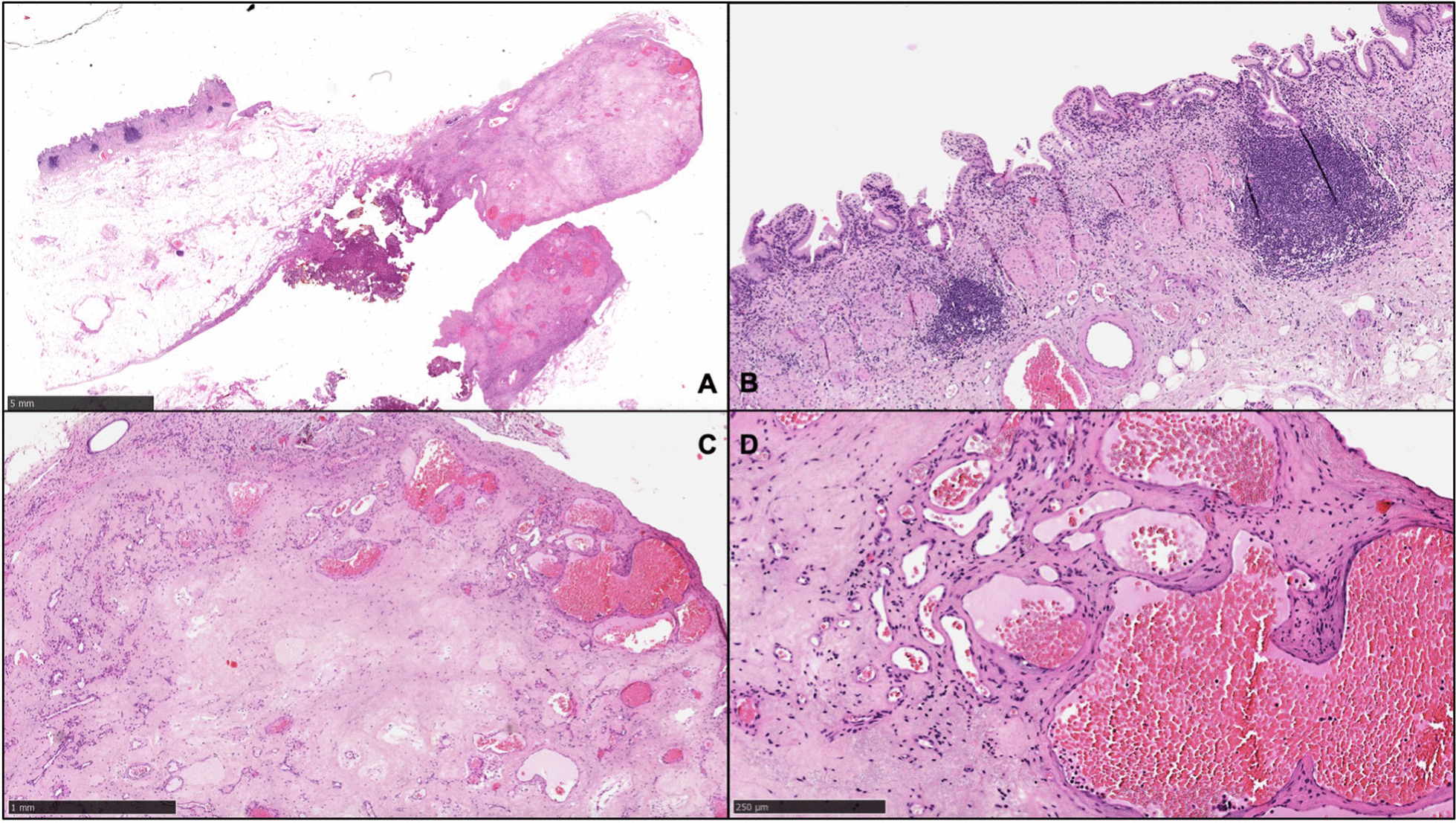
Histological features of the here reported gallbladder hamangioma. **A** Low power (H&E 20X) image of the 0.8 cm nodule within the gallbladder fundus (arrow); **B** Gallbladder mucosa and submucosa showing chronic follicular inflammation (H&E 100X); **C** The nodule consisted of multiple dilated vascular channels lined by endothelial cells arranged in a lobular fashion and admixed with a diffuse sclerosing matrix (H&E 40X); **D** No cytological atypias or mitotic figures were observed, a finding consistent with a benign neoplasm (H&E 200X)

Random sampling of the gallbladder fundus and body showed chronic follicular inflammation, a finding consistent with chronic cholecystitis. Microscopical examination of the nodule showed multiple dilated vascular channels within a sclerosing matrix, encased in the gallbladder wall (Fig. 3). Diagnosis of a cavernous hemangioma with sclerosing features was thus made.

## Discussion

Gallbladder pathological conditions represent a wide spectrum of alterations including gallstones, the most common of all digestive diseases [4]. In comparison, other gallbladder pathologies are significantly rarer [5–7], even though some, such as gallbladder and biliary tract cancer, represent a major health burden worldwide [8]. Gallbladder hemangioma represents an exceedingly rare entity with only ten cases described in literature hitherto (Table 1 and Fig. 1) [9–18]. We here report the first case of a gallbladder hemangioma coexisting with gallstones.

**Table 1:** Summary of gallbladder hemangiomas reported in literature

Reference	Age	Gender	Symptoms	Investigation	Pre-operative findings	Pre-operative diagnosis	Operation performed	Intra-operative findings	Size (cm)	Histopathological diagnosis
PMID: 6020957	60	M	Crampy epigastric pain	IV cholangiography	None	Post- cholecystectomy syndrome	Resection	Lobulated hemangiomatous mass in the gallbladder fossa	8 × 6	Cavernous hemangioma
PMID: 5793686	43	M	Vague abdominal pain	Oral cholecystogram	Intraluminal defect	Benign tumor of the fundus	Cholecystectomy	Purple tumor on the serosal surface of fundus	2 × 1	Cavernous hemangioma
PMID: 4689894	57	M	Crampy epigastric pain and obstructive jaundice	Oral cholecystogram	Non-functioning gallbladder	Cholecystitis, cholelithiasis, choledocholithiasis	Subtotal cholecystectomy	Enlarged gallbladder with intravisceral arterial bleeding associated to a gallbladder hemangioma involving the right side of the liver	12 × 5	Cavernous hemangioma
PMID: 594053	62	M	Symptoms suggestive for hepatic abscess	Post-mortem autopsy	Not applicable	Not applicable	Not applicable	Not applicable	Not reported	Cavernous hemangioma
PMID: 3305129	11	F	Pain in the right hypochondriac region	X-Ray, US, arteriography	Hyperechoic, lobular mass with phleboliths in lower right hepatic lobe	Cavernous hemangioma of the liver	Cholecystectomy	Large hemangioma involving the gallbladder posterior wall and hilum	8 × 7	Venous hemangioma
PMID: 9129455	56	M	Vague back pain	CT	Enhanced mass in gallbladder tail	Gallbladder adenomyomatosis vs adenocarcinoma	Extended cholecystectomy	White nodule in the gallbladder fundus within the hepatic bed	2 × 0.8	Arteriovenous hemangioma
PMID: 9065585	50	M	Pain in the right hypochondriac region, fever, nausea	CT	Large muticystic intraperitoneal mass extending from the subhepatic region to the right iliac fossa No gallbladder detected	Cystic mesenteric tumor	Cholecystectomy	Significant enlargement of gallbladder	10 × 0.9 × 6	Cavernous hemangioma
PMID: 16459643	49	F	None	US, CT	Echogenic, mildly enhanced mass of gallbladder fundus. Suspected invasion of the V liver segment	Gallbladder adenomyomatosis vs adenocarcinoma	Laparoscopic cholecystectomy	Purple lobulated mass within the gallbladder fundus wall involving the serosal surface with adhesion to liver	3	Cavernous hemangioma
https://doi.org/10.9738/INTSURG-D-15-00015.1	51	F	None	Endoscopic US, CT	Slightly hyperdense homogeneous and not vascularized mass on gallbladder fundus	Gallbladder adenomyomatosis or submucosal tumor	Laparoscopic cholecystectomy	Dark-brown nodular lesion protruding from the gallbladder fundus	1.8 × 1.7	Cavernous hemangioma
PMID: 31123450	75	M	None	CT, MRI	Calcified mass in the gallbladder	Gallbladder carcinoma	Extended cholecystectomy	Hardened and thickened gallbladder wall, adherent to the duodenum, transverse colon and bile duct	10 × 8 × 5.5	Cavernous hemangioma
Present case	76	M	Heartburn and abdominal pain due to the coexistent cholelithiasis and cholecystitis	X-Ray and US	Multiple gallstones, a thickened gallbladder wall with mild edema of the perivisceral adipose tissue and a 12 mm hypoechogenic area in the liver consistent with a hepatic angioma	Cholelithiasis and cholecystitis	Laparoscopic cholecystectomy	Subserosal nodule within the gallbladder fundus	0.8 × 0.5	Cavernous hemangioma

Overall, gallbladder hemangiomas have been reported to occur at a mean age of 54 ± 17 years old [range: 11–76] and predominantly in males (8/11) [9–18]. Interestingly, females tend to be younger at diagnosis than males (37 ± 23 years old *vs*. 60 ± 11 years old) [9–18]. Our patient is the oldest patient ever reported with this lesion.

Clinically, gallbladder hemangiomas tend to present with poorly localized abdominal pain, and often mimic other gallbladder disorders such as choledocholithiasis, cholelithiasis and cholecystitis [9–18] (Fig. 1). Whereas the symptomatology of previous reported cases has been ascribed to the mass effect exerted by the lesion [9–18], especially when of large dimensions, in our case the presence of gallstones justified the clinical presentation.

In line with the current guidelines, abdominal ultrasound was performed as a first-level exam [19], revealing multiple gallstones and a thickened gallbladder wall with mild edema of the perivisceral adipose tissue (Fig. 2). Clinical and radiological findings were suggestive of cholelithiasis with cholecystitis, and thus a cholecystectomy was planned.

However, during the surgical resection a subserosal nodule was detected in the gallbladder fundus, prompting a histopathological examination of this unexpected finding. In the present case, imaging failed to identify this nodule. This possibility is well-acknowledged and can be due to multiple factors related both to the operator and patient's characteristics like body habitus. It should also be noted that imaging of intramural lesions is so far unable to reliably distinguish an early stage gallbladder malignancy from benign conditions [20], thus, even if it had been detected, surgical resection followed by histopathological examination would have been the correct diagnostic/therapeutic approach. Considering the difficulties in detecting small intramural gallbladder lesions by ultrasound imaging, the incidence of gallbladder hemangiomas is probably underestimated, but novel techniques like high-resolution ultrasound can enhance the detection of smaller lesions, also improving the evaluation of gallbladder wall layers and helping distinguish between benign and malignant conditions [21].

Macroscopically, gallbladder hemangiomas tend to present as a purplish bosselated mass encased within the gallbladder wall and protruding inside the gallbladder lumen and/or from the serosal surface (Fig. 1). Although benign, these lesions can reach remarkable dimensions (up to 12 cm) and extend to the entire gallbladder. Other than the present, only one of the previously reported cases also presented as a well-defined grayish-whitish nodule on the serosal surface (Table 1 and Fig. 1).

At microscopical examination, gallbladder hemangiomas typically present as a mass of multiple enlarged, dilated vascular channels lined by endothelial cells and arranged in a lobular pattern, without atypia or mitoses [16, 18]. In our case, prominent sclerosis was also present, suggesting a long-standing lesion (Fig. 3).

Regarding tumor location, the here reported gallbladder hemangioma was found in the fundus, a finding in line with previously described cases [9–18] (5/11, 45%; Fig. 1). The pathophysiological mechanism leading to hemangioma development in this peculiar site is still unclear, but it is known that an hypoxic environment, such as the one in the relatively poorly vascularized gallbladder fundus, may lead to an upregulation of hypoxia inducible factor-1*α* (HIF-1*α*)-responsive chemokines such as stromal cell derived factor-1*α* (SDF-1*α*) and vascular endothelial growth factors (VEGF), both of which promote the recruitment and proliferation of endothelial progenitor cells [22]. The preponderance of gallbladder hemangiomas sited within the fundus [9–18] (Table 1 and Fig. 1) may support the hypothesis that hemangiomas may be a result of a homeostatic attempt to revascularize a relatively hypoxic tissue [23].

On the other hand, the frequent co-existence of gallbladder hemangiomas with additional angiomatous lesions, in multiple [12] or in a single other organ such as the vocal cords [10] or the liver [11, 16] (Fig. 1), challenges the aforementioned hypothesis and may suggest a common predisposition for the development of these lesions. In our case, the patient also presented a hepatic angioma (Fig. 2), identified by the abdominal ultrasound.

## Conclusions

The here described case is the first report of a gallbladder hemangioma coexisting with gallstones. Gallbladder hemangioma represents a rare, probably underreported entity which can be undetected. A correct estimation of the incidence of gallbladder hemangiomas could help shed a light on the underlying pathophysiological mechanisms, which are still unclear.

## Data Availability

Data sharing is not applicable to this article as no datasets were generated or analyzed during the current study.

## Abbreviations

HIF-1*α*: Hypoxia inducible factor-1*α*
SDF-1*α*: Stromal cell derived factor-1*α*
VEGF: Vascular endothelial growth factor
